# Intestinal Perforation Secondary to Mucormycosis Associated With Puerperal Sepsis

**DOI:** 10.7759/cureus.17428

**Published:** 2021-08-25

**Authors:** Vivek Bhat, Anitha S, Anu Thomas, Jayashree V Kanavi, Annamma Thomas

**Affiliations:** 1 Obstetrics and Gynecology, St. John's Medical College, Bangalore, IND

**Keywords:** immunocompetent, fungal infection, pregnant, postpartum, peritonitis

## Abstract

Mucormycosis is a rare opportunistic infection, usually seen in diabetics, immunocompromised, or those with coronavirus disease 2019 (COVID-19). Gastrointestinal involvement is uncommon but often deadly. We report a case of gastrointestinal mucormycosis causing intestinal perforation in a non-diabetic, COVID-19 negative, immunocompetent woman, associated with puerperal sepsis.

A 22-year-old woman presented to our center on post-natal day five, following delivery with insertion of an intrauterine contraceptive device (IUCD). She had complaints of breathlessness, fatigue, and giddiness. Examination revealed tachycardia, tachypnea, hypotension, and bilateral pedal edema. Following appropriate investigations, she was diagnosed with puerperal sepsis with pre-renal acute kidney injury. Imaging was suggestive of retained products of conception, and she subsequently underwent dilation and evacuation (D&E) on day eight of admission. Following brief symptomatic improvement, on day 10 of admission, she developed vomiting, abdominal distension, and pain, with obstipation. Erect X-ray showed air under the diaphragm, suggestive of perforation. She emergently underwent laparotomy with limited right hemicolectomy, ileostomy with mucous fistula. Intraoperative findings revealed a closed-loop obstruction involving terminal ileum, with two perforations. The biopsy report later revealed colonization of Mucor and hemorrhagic necrosis along the entire length of the resected specimen. She was started on amphotericin B, and after a slow recovery, was discharged.

Gastrointestinal mucormycosis is rare and has a mortality rate of 94%. It is usually seen in those with predisposing factors for mucormycosis. This is the first report of mucormycosis associated with puerperal sepsis. It is typically acquired via ingestion and may cause perforation, where mortality is further increased. Diagnosis can only be confirmed by histopathology demonstrating the characteristic morphology of Mucor. Treatment requires resection of necrotic tissues, intensive treatment with amphotericin B, and correction of predisposing factors. Our case highlights the need for a high degree of suspicion for mucormycosis in patients with intestinal perforation, even if immunocompetent, and its potential association with puerperal sepsis.

## Introduction

Mucormycosis is a rare opportunistic infection caused by fungi of the order *Mucorales* [1]. Common risk factors include diabetes mellitus, immunosuppression due to hematologic malignancies and chemotherapy, iron overload [2], and in recent times, particularly in India, infection with coronavirus disease 2019 (COVID-19) [3]. Contributory factors that allow fungal proliferation include decreased phagocytes or phagocyte function, or excessive free iron in the host [4]. While the most common presentation is rhino-orbito-cerebral mucormycosis, it can rarely affect the gastrointestinal tract (GIT). Due to nonspecific symptoms, and the resultant diagnostic delay, the prognosis in these cases is often poor [2]. We report an immunocompetent, non-diabetic, COVID-19 negative patient with ileal perforation due to gastrointestinal mucormycosis associated with puerperal sepsis.

## Case presentation

A 22-year-old woman with an obstetric score of para 2, living 2 (P2L2) underwent a full-term vaginal delivery with episiotomy at another center. She delivered a healthy baby, following which, on the same day, an intrauterine contraceptive device (IUCD) was inserted; she was later discharged. On post-natal day five, she suddenly developed breathlessness, fatigue, and giddiness. At this point, she was brought to our center.

Examination revealed tachycardia (heart rate - 138/minute), tachypnea (respiratory rate - 44/min), hypotension (systolic blood pressure - 70 mmHg), and bilateral pedal edema, with an oxygen saturation of 94%. Local examination revealed foul-smelling vaginal discharge and a gaping episiotomy wound. Her investigations are summarised in Table 1.

**Table 1:** Investigative values at admission. TLC - total leukocyte count; DC - differential count; ESR - erythrocyte sedimentation rate; INR - international normalized ratio; aPTT - activated partial thromboplastin time; AST - aspartate transaminase; ALT - alanine transaminase; ALP - alkaline phosphatase; GGT - gamma glutamyl transferase; LDH - lactate dehydrogenase.

Investigation	Patient value	Normal value
Hemoglobin	9.50%	12-16%
TLC	20,780/uL	4,000-11,000
DC	88% Neutrophil, 4% Leukocyte	40-75%, 20-45%
Platelets	178,000/uL	150,000-400,000/uL
ESR	12 seconds	0-20 seconds
INR	1.6	0.8-1.1
aPTT	37.20	26-35 seconds
D-dimer	3536 ng/mL	0-255 ng/mL
Procalcitonin	5.73 ng/mL	0-0.5 ng/mL
Urea	90.1 mg/dL	19.0-44.0 mg/dL
Creatinine	1.24 mg/dL	0.72-1.25 mg/dL
AST	17 U/L	5-34 U/L
ALT	13 U/L	5-34 U/L
ALP	139 U/L	48-95 U/L
GGT	43 U/L	9-36 U/L
LDH	253 U/L	130-250 Y/L
Sodium	139 mEq/L	136-145 mEq/L
Potassium	3.3 mEq/L	3.5-5.1 mEq/L

In view of shock, she was intubated and shifted to the intensive care unit (ICU), and started on inotropes. Vaginal toileting was done, and a wound swab was sent. Emergency lower limb Doppler scan revealed no deep vein thrombosis. Her potassium was corrected, and a diagnosis of puerperal sepsis with pre-renal acute kidney injury was made.

**Figure 1:**
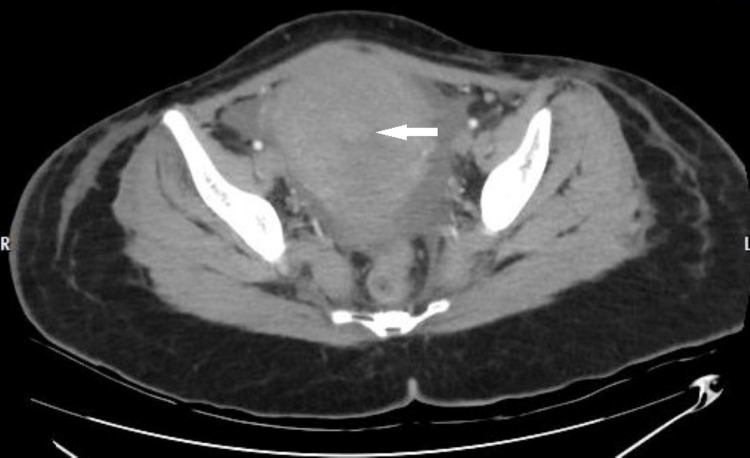
CECT pelvis showing a bulky uterus with heterogenous hyperdense content in the endometrial cavity, suggestive of retained products of conception, and no evidence of IUCD. CECT - contrast-enhanced computed tomography; IUCD - intra-uterine contraceptive device.

On post-natal day six (day two of admission), she developed four spikes of fever, ranging from 102˚C to 104˚C. In view of fever, tests for malaria and dengue, Weill Felix test, Widal test for typhoid, and a reverse transcription-polymerase chain reaction (RT-PCR) for COVID-19 were all performed; they all came negative. Wound and cervical swabs later showed *Staphylococcus aureus*, with blood cultures showing no growth. Based on sensitivity results, she was started on meropenem, vancomycin, metronidazole, and colistin. Her total leucocyte count ranged from 20,000 to 25,000/uL, her platelet count ranged from 75,000 to 247,000/uL, and her creatinine improved to 0.60mg/dL. Contrast-enhanced computed tomography (CECT) of the pelvis showed a bulky uterus with heterogenous hyperdense content in the endometrial cavity with dimensions 3 x 4 x 5 cm, suggestive of retained products of conception, with no evidence of IUCD (Figure 1).

She subsequently underwent dilation and evacuation (D&E) under general anesthesia. On post-natal day 12 (day eight of admission), she was extubated, and then shifted out from the ICU.

**Figure 2:**
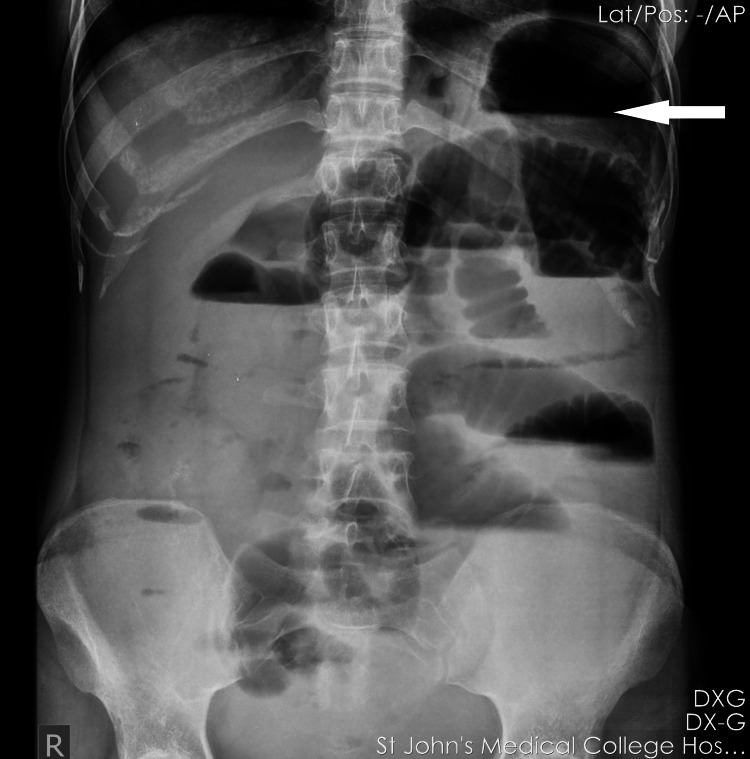
Erect X-ray showing air under the diaphragm, suggestive of gastrointestinal perforation.

She briefly improved symptomatically, but two days later, on day 10 of admission, she developed vomiting, abdominal distension, and pain, with obstipation. Erect X-ray showed air under the diaphragm, suggestive of gastrointestinal perforation (Figure 2).

Ultrasound abdomen revealed pneumoperitoneum with septated ascites, hollow viscus perforation, and dilated bowel loops. She underwent an emergency laparotomy, followed by a limited right hemicolectomy, ileostomy with mucous fistula. Intraoperative findings revealed a closed-loop obstruction involving terminal ileum, gangrene of terminal ileum for about 40 cm length involving the ileocecal junction and proximal 5 cm of the cecum. The terminal ileum had two perforations, and approximately 60 mL of pus had collected in the right iliac fossa.

**Figure 3:**
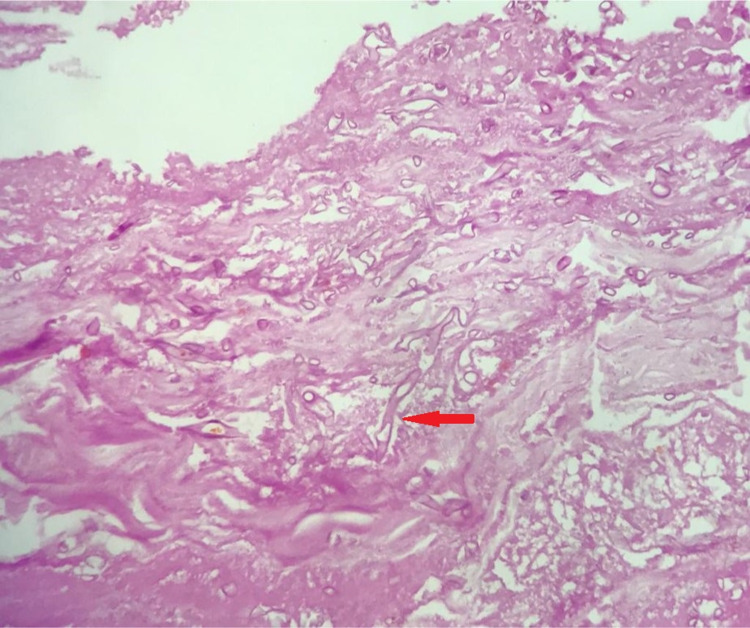
Histopathology specimen showing hyphae of Mucor.

On postoperative day two (day 12 of admission), she developed a burst abdomen, which was conservatively managed. The biopsy later revealed mucosal and serosal colonization of Mucor along the entire length of the resected specimen, along with hemorrhagic necrosis (Figure 3).

She was immediately started on colistin and amphotericin B, and vacuum-assisted closure was done in two sittings for burst abdomen. After a slow recovery, she was discharged with one month of amphotericin B and advised daily dressing of her surgical site.

## Discussion

Fungi of the order *Mucorales* are ubiquitous in nature. Infection i.e. mucormycosis is most commonly due to Rhizopus species. Pathologically, mucormycosis is characterized by aggressive angioinvasion, causing tissue necrosis [5]. While rare, mucormycosis is being increasingly reported [6]. In India, diabetes mellitus remains the most common factor [2], with COVID-19 gaining notoriety in recent times [3]. GIT involvement is seen in less than 10% of all patients [2]. It is uncommon in the immunocompetent. Further, it is more commonly reported in the pediatric population [2]. Our patient was an adult postpartum woman with no traditional risk factors, including COVID-19.

Gastrointestinal mucormycosis is typically acquired via ingestion. The stomach is most commonly involved, followed by the colon and the ileum. Symptoms vary according to the site involved but are nonspecific. Abdominal pain and distension, associated with nausea and vomiting are the most common [6]. It may present as an abdominal mass or perforation [5]. The mortality is high, up to 94% in India [2,5,7]. This is particularly increased by perforation [7].

In India, gastrointestinal perforation is usually due to infectious causes, such as tuberculosis, typhoid, and *Helicobacter pylori* infections [8]. Mucormycosis is rarely suspected, leading to diagnostic delay.

Due to its high mortality, rapid diagnosis and treatment are essential. Imaging findings in the appropriate context may be suggestive - in small bowel mucormycosis, as in our case, the findings are those of multifocal ischemia [9]. However, the mainstay of diagnosis remains the histopathological examination of biopsy specimens, with or without polymerase chain reaction (PCR), which classically shows non-septate hyphae branching at right angles [10]. Treatment requires three main pillars. The first is excision of the necrotic tissues - in our case, resection and anastomosis of the affected bowel segment. The second is appropriate, aggressive medical treatment. Amphotericin B is the first line drug, followed by posaconazole or isavuconazole [2]. Early initiation of drugs, in addition to appropriate surgical debridement, improves prognosis [10]. The final pillar is correcting the predisposing conditions for infection. In the case of puerperal sepsis, a challenging condition on its own, this entails rapid identification of shock, broad-spectrum antibiotics, and management of organ dysfunction by a multidisciplinary team [11].

To our knowledge, this is the first case of gastrointestinal mucormycosis associated with puerperal sepsis in English literature. Our patient developed abdominal symptoms after the improvement of septic manifestations. With appropriate surgery and antifungal therapy, she recovered well.

While pregnancy and the postpartum period have an increased risk of infections such as tuberculosis, due to complex changes in the immune system to prevent allograft rejection of the fetus [12], no predisposition to mucormycosis has been reported. Our patient’s lymphocyte counts remained elevated throughout her illness, ruling out lymphopenia as a cause. Similarly, while antibiotic therapy can lead to gastrointestinal infections such as *Clostridium difficile* [13], no such association has been reported for mucormycosis. It is possible that our patient had silent mucormycosis before developing puerperal sepsis, although we cannot prove this.

## Conclusions

The gastrointestinal tract is a rare site of infection in mucormycosis. It may present as intestinal perforation, even in those without traditional risk factors for mucormycosis, such as diabetes mellitus, COVID-19, or immunocompromise. These cases tend to have a poor prognosis, so, in such patients, a high degree of suspicion for mucormycosis is needed. Rapid action is needed for the perforation to avoid a fatal outcome. After histopathological confirmation of mucormycosis, therapy with amphotericin B and correction of the underlying factors for mucormycosis are needed. Finally, our case highlights the potential association of puerperal sepsis with mucormycosis.

## References

[1] Hibbett DS, Binder M, Bischoff JF (2007). A higher-level phylogenetic classification of the fungi. Mycol Res.

[2] Prakash H, Chakrabarti A (2021). Epidemiology of mucormycosis in India. Microorganisms.

[3] Sen M, Honavar SG, Bansal R (2021). Epidemiology, clinical profile, management, and outcome of COVID-19-associated rhino-orbital-cerebral mucormycosis in 2826 patients in India - Collaborative OPAI-IJO Study on Mucormycosis in COVID-19 (COSMIC), report 1. Indian J Ophthalmol.

[4] Ibrahim AS, Spellberg B, Walsh TJ, Kontoyiannis DP (2012). Pathogenesis of mucormycosis. Clin Infect Dis.

[5] Petrikkos G, Skiada A, Lortholary O, Roilides E, Walsh TJ, Kontoyiannis DP (2012). Epidemiology and clinical manifestations of mucormycosis. Clin Infect Dis.

[6] Spellberg B (2012). Gastrointestinal mucormycosis: an evolving disease. Gastroenterol Hepatol (N Y).

[7] Roden MM, Zaoutis TE, Buchanan WL (2005). Epidemiology and outcome of zygomycosis: a review of 929 reported cases. Clin Infect Dis.

[8] Hameed T, Kumar A, Sahni S, Bhatia R, Vidhyarthy AK (2020). Emerging spectrum of perforation peritonitis in developing world. Front Surg.

[9] Ghuman SS, Sindhu P, Buxi TB, Sheth S, Yadav A, Rawat KS, Sud S (2021). CT appearance of gastrointestinal tract mucormycosis. Abdom Radiol (NY).

[10] Farmakiotis D, Kontoyiannis DP (2016). Mucormycoses. Infect Dis Clin North Am.

[11] Buddeberg BS, Aveling W (2015). Puerperal sepsis in the 21st century: progress, new challenges and the situation worldwide. Postgrad Med J.

[12] Abu-Raya B, Michalski C, Sadarangani M, Lavoie PM (2020). Maternal immunological adaptation during normal pregnancy. Front Immunol.

[13] Song HJ, Shim KN, Jung SA (2008). Antibiotic-associated diarrhea: candidate organisms other than Clostridium difficile. Korean J Intern Med.

